# Supplementary material for: Single-cell DNA methylome and 3D multi-omic atlas of the adult mouse brain
Source: Nature. 2023 Dec 13;624(7991):366–77. doi: 10.1038/s41586-023-06805-y (PMC10719113; doi:10.1038/s41586-023-06805-y)

nature portfolio

https://doi.org/10.1038/s41586-023-06805-y

Supplementary information

Single-cell DNA methylome and 3D multi-omic atlas of the adult mouse brain

In the format provided by the authors and unedited

Supplementary Note 1. A detailed illustration of the Tle4 gene example in Fig. 1d

In this example, cells with a low mCH fraction in the *Tle4* gene body exhibit high RNA expression levels (Fig. 1d, left), achieving a strong negative correlation across 4,673 cell groups (Pearson correlation coefficient, PCC. -0.86, p-value < 10^{-5}, permutation test). Similarly, the mCG fraction of an example DMR located in the *Tle4*-upstream region negatively correlates with chromatin accessibility signals (PCC. -0.73, p-value < 10^{-5}, permutation test, Fig. 1d, right). Furthermore, the m3C dataset provides chromatin contact information, indicating physical proximity between DMRs and the *Tle4* gene in the “L6 CT CTX Glut” compared to the “Pvalb Gaba” subclass, where *Tle4* expression is low (Fig. 1d, middle). The base-resolution methylation profiles further reveal intricate cell-type specific epigenomic patterns, which offer rich information about the precise control of gene expression and transcript isoforms (Fig. 1d, bottom, and later section). The following link can visualize the Tle4 gene example interactively in our web application:
https://mousebrain.salk.edu/dynamic_browser?cemba_cell,continuous_scatter,mc_all_tsne,gene_mch:Tle4?cemba_cell,continuous_scatter,mc_all_tsne,gene_rna:Tle4?cemba_cell,categorical_scatter,mc_all_tsne,CellSubClass?higlass,multi_cell_type_2d,cell_types=L6 CT CTX Glut+Pvalb Gaba,region1=Tle4

Supplementary Note 2. Cell subclass global methylation level

The global mCG fractions for all cell groups span from 66.3%-85.3% (29.0-37.3 million CpG sites), while mCH fractions range between 0.6%-5.6% (6.9-59.2 million CpH sites). Many subcortical neuronal subclasses exhibit substantially elevated mCH levels compared to excitatory neurons in cortical regions (Extended Data Fig. 3i-j). Examples include "AD Serpinb7 Glut" (from TH, mCH level 5.6%), "PG-TRN-LRN Fat2 Glut" (HB, 5.4%), "CBN Glut" (CB, 5.4%), "SNr-VTA-PPN Pax5 Npas1 Gaba" (MB, 5.2%), “PM-TM-PVp Tbx3 Hist-Gaba” (HY, 4.5%). Since CpH sites (1.1×10^9) are more abundant than CpG sites (4.3×10^7) in the mouse genome, the mCH sites in these cells surpass the total number of CpG sites, highlighting the functional significance of this unique neuronal epigenetic modality[1,2].

Supplementary Note 3. Discussion about the GRN triples’ correlation combinations

We summarized eight possible correlation combinations into four models and one unknown category (Extended Data Fig. 11g). The most frequent model (39.8%) represents all positive correlations, indicating that both the TF and DMR have an active

effect on the gene (Model 1). The second most frequent model (30.5%) is negative for TF-Target and TF-DMR edges and positive for DMR-Target edges, suggesting that the TF plays a repressive role by repressing active DMRs. These two models account for most (70.3%) of the edges, indicating that intersected DMRs predominantly activate target genes. The third (Model 3, 11.1%) and fourth (Model 4, 11.1%) most popular models are negative or repressive for DMR-Target edges, with Model 3 suggesting that active TFs turn off repressive DMRs to activate genes and Model 4 indicating that repressive TFs turn on repressive DMRs to deactivate genes. The remaining edges are assigned to the "Unknown" group (Extended Data Fig. 11f), likely intersected by chance or representing indirect relationships involving additional regulatory factors. Models 1 to 4 cover 92.5% of the edges, demonstrating a remarkable correspondence between these three genomic elements among the brain-wide cell types.

Supplementary Note 4. More examples of GRN triples

Here, we discuss some more examples of GRN triples. The first one is for the *Egr1* cofactor *Erf*[3], which is also connected to *Nab2* through another DMR (Extended Data Fig. 12a, b). Another interesting edge connects *Egr1* with the *Synpo* gene, which encodes an actin-associated postsynaptic protein, with a DMR located in its upstream correlated regions (Extended Data Fig. 12c, d). A third example is the link between the subcortical expressing TF *Stat5b* and the *Cacna2d2* gene, which encodes a calcium voltage-gated channel auxiliary subunit connected by an intragenic DMR located in the highly correlated gene body domain (Extended Data Fig. 12e, f).

Supplementary Note 5. The PageRank score of the Rfx gene family

The *Rfx* gene family[4] has six members variably expressed in adult mouse brains. Their connectivity on the GRN and subclass-specific PageRank scores reveal that these members could play distinct regulatory roles that partially overlap (Extended Data Figure 12i). For example, *Rfx2* is predicted to be critical in HY and MB cell types. *Rfx3* has high PageRank scores in cell types overlapping with *Rfx2* in subcortical areas but is also inferred as an important regulator broadly in cortical excitatory neurons. *Rfx5* is predicted to be important in a wider range of cell types, including the majority of subcortical neurons, cortical inhibitory neurons, astrocytes, and oligodendrocyte progenitors.

Supplementary Note 6. Genomic regions show associations between epigenetic signals and isoform diversity.

Here, we show two examples of genes whose alternative usage is likely under epigenetic regulation. The first example plots the genome browser view of alpha and beta-*Nrxn3* promoters (Extended Data Fig. 13e) in five representative cell types. The canonical alpha-*Nrxn3* promoter has a low expression in the "TH Prkcd Grin2c Glut" subclass, evidenced by high mCG and mCH fractions downstream of the promoter. In contrast, the beta-promoter shows the highest expression level in the "TH Prkcd Grin2c Glut" subclass, with depleted surrounding methylation. Intriguingly, the transcript body domain of beta-*Nrxn3* exhibits associated interaction changes among cell types with different beta-*Nrxn3* expression (Extended Data Fig. 13e). Similarly, the first exon (ENSMUSE00000683442) of the longest *Oxr1* transcript displays increased usage PSI among representative cell subclasses, accompanied by corresponding methylation and chromatin conformation changes in the surrounding regions (Extended Data Fig. 13f).

1. Clemens, A. W. & Gabel, H. W. Emerging Insights into the Distinctive Neuronal Methylome. *Trends Genet.* (2020) doi:10.1016/j.tig.2020.07.009.
2. He, Y. & Ecker, J. R. Non-CG Methylation in the Human Genome. *Annu. Rev. Genomics Hum. Genet.* **16**, 55–77 (2015).
3. Tarcic, G. *et al.* EGR1 and the ERK-ERF axis drive mammary cell migration in response to EGF. *FASEB J.* **26**, 1582–1592 (2012).
4. Sugiaman-Trapman, D. *et al.* Characterization of the human RFX transcription factor family by regulatory and target gene analysis. *BMC Genomics* **19**, 181 (2018).

Supplementary Note 7. snmC and snm3C-seq sample FANS gating examples

snm3C-seq FANS gating example

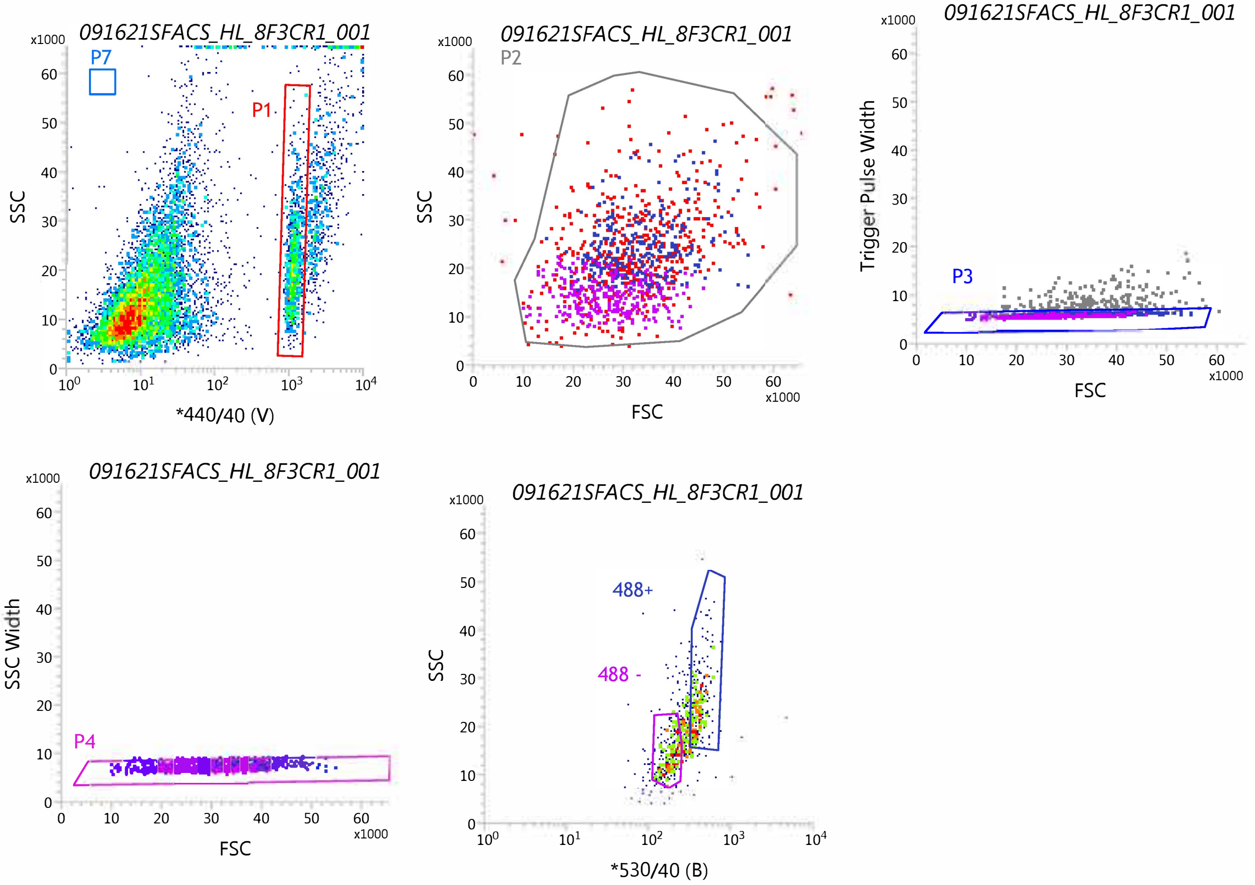

Statistics: *091621SFACS_HL_8F3CR1_001*			
Populations	Events	% Total	% Parent
All Events	10,140	100.00%	####
P1	1,049	10.35%	10.35%
P2	1,035	10.21%	98.67%
P3	803	7.92%	77.58%
P4	752	7.42%	93.65%
488+	277	2.73%	36.84%
488 -	267	2.63%	35.51%

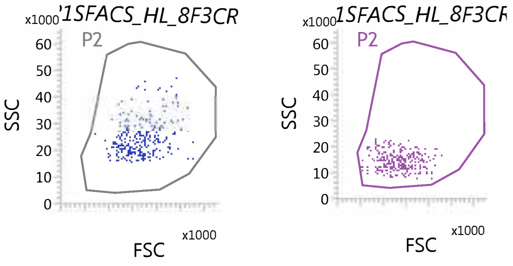

snmC-seq3 FANS gating example

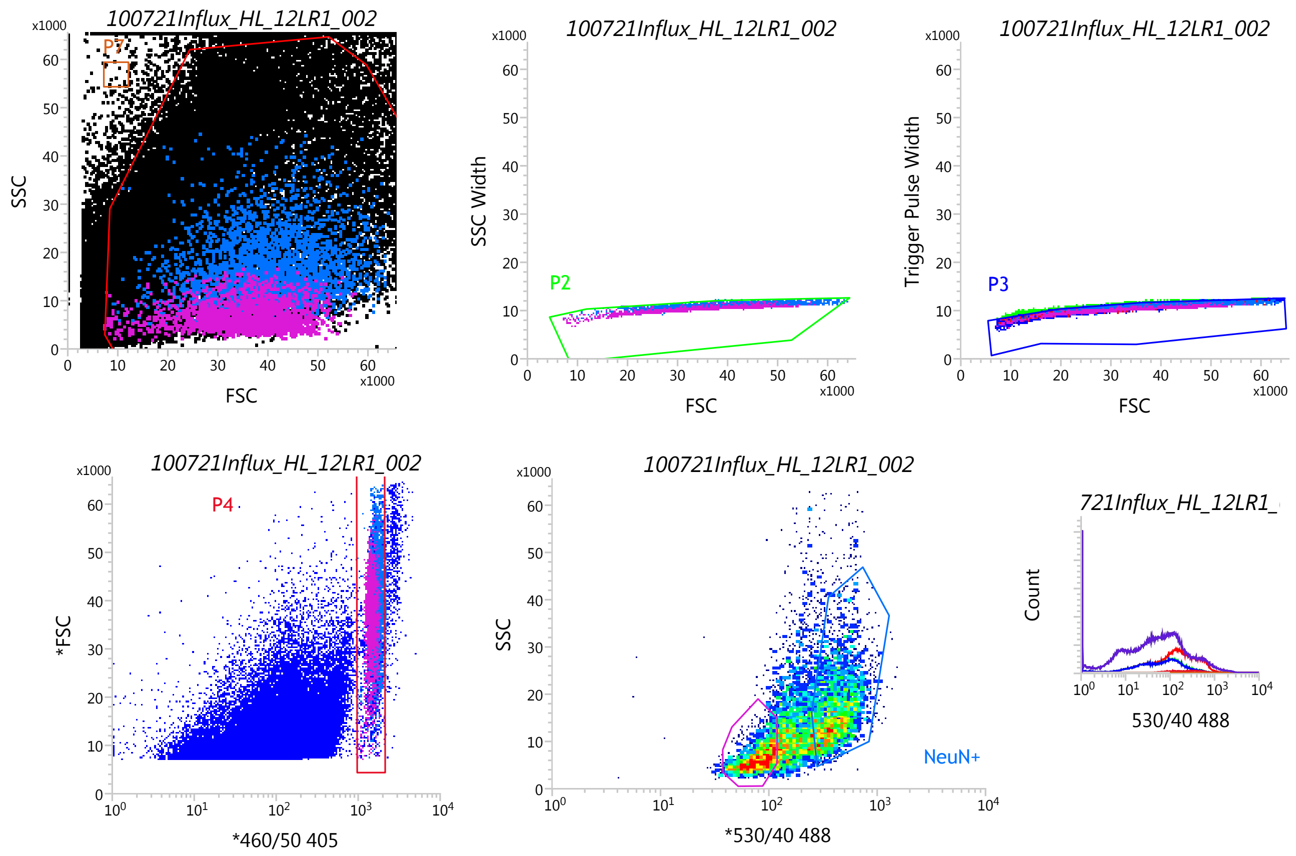

Populations: *100721Influx_HL_12LR1_002*

Populations	Events	% Total	% Parent
All Events	284,075	100.00%	####
P1	116,722	41.09%	41.09%
P2	69,020	24.30%	59.13%
P3	66,582	23.44%	96.47%
P4	7,324	2.58%	11.00%
NeuN+	2,930	1.03%	40.01%
Neg	1,901	0.67%	25.96%
P7	17	0.01%	0.01%
NOT(P7)	284,058	99.99%	99.99%

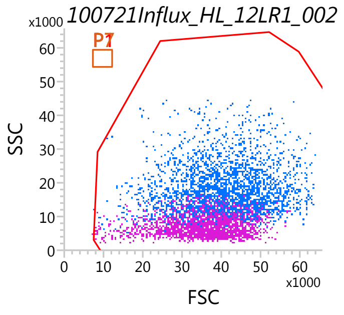

Supplement: Supplementary file 1 — Supplementary Notes 1–7, containing additional details to the text and data presented in the main text and methods section. [file 41586_2023_6805_MOESM1_ESM.pdf]
