# Supplementary material for: Single-cell DNA methylome and 3D multi-omic atlas of the adult mouse brain
Source: Nature. 2023 Dec 13;624(7991):366–77. doi: 10.1038/s41586-023-06805-y (PMC10719113; doi:10.1038/s41586-023-06805-y)
Supplement: Supplementary file 2 — Reporting Summary [file 41586_2023_6805_MOESM2_ESM.pdf]

nature portfolio

Corresponding author(s): Joseph R. Ecker

Last updated by author(s): Oct 29, 2023

Reporting Summary

Nature Portfolio wishes to improve the reproducibility of the work that we publish. This form provides structure for consistency and transparency in reporting. For further information on Nature Portfolio policies, see our Editorial Policies and the Editorial Policy Checklist.

Statistics

For all statistical analyses, confirm that the following items are present in the figure legend, table legend, main text, or Methods section.

n/a	Confirmed	
☐	☒	The exact sample size (*n*) for each experimental group/condition, given as a discrete number and unit of measurement
☐	☒	A statement on whether measurements were taken from distinct samples or whether the same sample was measured repeatedly
☐	☒	The statistical test(s) used AND whether they are one- or two-sided<br>*Only common tests should be described solely by name; describe more complex techniques in the Methods section.*
☐	☒	A description of all covariates tested
☐	☒	A description of any assumptions or corrections, such as tests of normality and adjustment for multiple comparisons
☐	☒	A full description of the statistical parameters including central tendency (e.g. means) or other basic estimates (e.g. regression coefficient) AND variation (e.g. standard deviation) or associated estimates of uncertainty (e.g. confidence intervals)
☐	☒	For null hypothesis testing, the test statistic (e.g. *F*, *t*, *r*) with confidence intervals, effect sizes, degrees of freedom and *P* value noted<br>*Give P values as exact values whenever suitable.*
☒	☐	For Bayesian analysis, information on the choice of priors and Markov chain Monte Carlo settings
☒	☐	For hierarchical and complex designs, identification of the appropriate level for tests and full reporting of outcomes
☐	☒	Estimates of effect sizes (e.g. Cohen's *d*, Pearson's *r*), indicating how they were calculated

Our web collection on statistics for biologists contains articles on many of the points above.

Software and code

Policy information about availability of computer code

Data collection	BD Influx Sortware v1.2.0.142 (flow cytometry), Freedom EVOware v2.7 (library preparation), Illumina MiSeq control software v3.1.0.13 and NovaSeq 6000 control software v1.6.0/RTA v3.4.4 (sequencing), Olympus cellSens Dimension 1.8 (image acquisition)
Data analysis	cemba-data, v1.6.8; cutadapt, v2.10; bismark, v0.20; bowtie2, v2.3; samtools, v1.9; Picard, v3.0.0;<br><br>Mapping pipeline for snmC-seq3 and snm3C-seq is available at https://hq-1.gitbook.io/mc/. Single-cell DNA methylome data analysis tools are available at ALLCools (v1.0.8) python package, https://lhqing.github.io/ALLCools/intro.html; Single-cell chromatin conformation data analysis tools are available at the scHiCluster (v1.3.2) python package, https://github.com/zhoujt1994/scHiCluster. Other codes and Jupyter Notebooks related to results and method sections are shared in this GitHub repository: https://github.com/lhqing/wmb2023.<br>Brain dissection 3D image created by ITK-SNAP (v4.0.0)<br>MERSCOPE data analysis were performed with MERSCOPE Instrument Software (v2023-01)

For manuscripts utilizing custom algorithms or software that are central to the research but not yet described in published literature, software must be made available to editors and reviewers. We strongly encourage code deposition in a community repository (e.g. GitHub). See the Nature Portfolio guidelines for submitting code & software for further information.

Data

Policy information about availability of data

All manuscripts must include a data availability statement. This statement should provide the following information, where applicable:

- Accession codes, unique identifiers, or web links for publicly available datasets
- A description of any restrictions on data availability
- For clinical datasets or third party data, please ensure that the statement adheres to our policy

The snmC-seq2/3 single-cell sequencing data and MERFISH dataset are accessible through the Neuroscience Multi-omic Data (NeMO) Archive (https://assets.nemoarchive.org/dat-sig83t9). The snm3C-seq single-cell sequencing data are accessible through NeMO (https://assets.nemoarchive.org/dat-sig83t9) and GEO (GSE213262). The MERFISH dataset will be accessible through GEO. The whole-brain snATAC-seq dataset is shared by Zu et al11. The whole-brain scRNA-seq MERFISH, and SMART-seq dataset is shared by Yao et al6. All the processed data related to results and method sections are shared in this GitHub repository: https://github.com/lhqing/wmb2023. Allen Brain Reference Atlas and Common Coordinate Framework is accessed through Wang et al3. A detailed description of the data availability is provided at https://mousebrain.salk.edu/download.

Human research participants

Policy information about studies involving human research participants and Sex and Gender in Research.

Reporting on sex and gender	This study did not involve human research participants.
Population characteristics	This study did not involve human research participants.
Recruitment	This study did not involve human research participants.
Ethics oversight	This study did not involve human research participants.

Note that full information on the approval of the study protocol must also be provided in the manuscript.

Field-specific reporting

Please select the one below that is the best fit for your research. If you are not sure, read the appropriate sections before making your selection.

☒ Life sciences ☐ Behavioural & social sciences ☐ Ecological, evolutionary & environmental sciences

For a reference copy of the document with all sections, see nature.com/documents/nr-reporting-summary-flat.pdf

Life sciences study design

All studies must disclose on these points even when the disclosure is negative.

Sample size	No statistical methods were used to predetermine sample size. We empirically determined to use two to three biological experiments for all single-cell epigenomic experiments to achieve minimum reproducibility for this large-scale project. For snmC-seq, at least 3,072 nuclei (eight 384-well plates) from each dissected region (1,536 nuclei from each replicate). For snm3C-seq, at least 6,144 nuclei (eight 384-well plates) from each dissected region (3,072 nuclei from each replicate). The sample size allowed us to obtain high coverage methylome and 3D genome for thousands of brain cell clusters, and perform confident downstream analyses.
Data exclusions	Primary quality control for DNA methylome cells was (1) overall mCCC level < 0.05; (2) overall mCH level < 0.2; (3) overall mCG level > 0.5; (4) total final reads > 500,000 and < 10,000,000; and (5) Bismarck mapping rate > 0.5. Additionally, we calculated lambda DNA spike-in methylation levels to estimate each sample's non-conversion rate. All samples demonstrated a low non-conversion rate (< 0.01). For the 3C modality in snm3C-seq cells, we also required cis-long-range contacts (two anchors > 2500 bp apart) > 50,000.
Replication	Each dissected region is represented by 2-3 replicates, obtained from pooling the same region from at least six animals. All replications were successful.
Randomization	Randomization is not applicable, since the cells collected are random by nature.
Blinding	Blinding is not applicable, since all data are collected from male C57BL/6J mice at the age of P56 when generating this reference brain atlas.

Reporting for specific materials, systems and methods

We require information from authors about some types of materials, experimental systems and methods used in many studies. Here, indicate whether each material, system or method listed is relevant to your study. If you are not sure if a list item applies to your research, read the appropriate section before selecting a response.

Materials & experimental systems

n/a	Involved in the study
☐	☒ Antibodies
☒	☐ Eukaryotic cell lines
☒	☐ Palaeontology and archaeology
☐	☒ Animals and other organisms
☒	☐ Clinical data
☒	☐ Dual use research of concern

Methods

n/a	Involved in the study
☒	☐ ChIP-seq
☐	☒ Flow cytometry
☒	☐ MRI-based neuroimaging

Antibodies

Antibodies used: AlexaFluor488-conjugated anti-NeuN antibody (MAB377X, Millipore, A60, monoclonal, 1:500 dilution)

Validation: All antibodies have been previously published for use in immunohistochemistry and flow cytometry experiments. See vendor's page here: https://www.emdmillipore.com/US/en/product/Anti-NeuN-Antibody-clone-A60-Alexa-Fluor488-conjugated,MM_NF-MAB377X

Animals and other research organisms

Policy information about studies involving animals; ARRIVE guidelines recommended for reporting animal research, and Sex and Gender in Research

Laboratory animals: Adult (P56) C57BL/6J male mice were purchased from Jackson Laboratories at seven weeks of age and maintained in the Salk animal barrier facility on 12-hour dark-light cycles with food ad-libitum for up to 10 days (Housing condition: Temperature: 21-23 C, relative humidity: 61-63%)

Wild animals: the study did not involve wild animals

Reporting on sex: We only used male mice, directly purchased from Jackson Laboratories. Sex difference was not considered in this study.

Field-collected samples: the study did not involve samples collected from the field

Ethics oversight: All experimental procedures using live animals were approved by the Salk Institute Animal Care and Use Committee under protocol number 18-00006.

Note that full information on the approval of the study protocol must also be provided in the manuscript.

Flow Cytometry

Plots

Confirm that:

☒ The axis labels state the marker and fluorochrome used (e.g. CD4-FITC).

☒ The axis scales are clearly visible. Include numbers along axes only for bottom left plot of group (a 'group' is an analysis of identical markers).

☒ All plots are contour plots with outliers or pseudocolor plots.

☒ A numerical value for number of cells or percentage (with statistics) is provided.

Methodology

Sample preparation: Isolated nuclei were labeled by incubation with 1:1000 dilution of AlexaFluor488-conjugated anti-NeuN antibody (MAB377X, Millipore) and a 1:1000 dilution of Hoechst 33342 at 4°C for 1 hour with continuous shaking. Fluorescence-Activated Nuclei Sorting (FANS) of single nuclei was performed using a BD Influx sorter with an 85µm nozzle at 22.5 PSI sheath pressure. Single nuclei were sorted into each well of a 384-well plate preloaded with 2 µl of Proteinase K digestion buffer (1µl M-Digestion Buffer, 0.1µl 20 µg/µl Proteinase K and 0.9µl H2O). The alignment of the receiving 384-well plate was performed by sorting sheath flow into wells of an empty plate and making adjustments based on the liquid drop position. Single-cell (1 drop single) mode was selected to ensure the stringency of sorting. For each 384-well plate, columns 1-22 were sorted with NeuN+ (488 +) gate, and column 23-24 with NeuN- (488-) gate, reaching an 11:1 ratio of NeuN+ to NeuN- nuclei.

Detail experiment protocol for snmC/snm3C-seq is provided in Supplementary Information 1

Instrument: BD Influx

Software	BD Influx Sortware v1.2.0.142
Cell population abundance	We sort NeuN+ (488+) gate and NeuN- (488-) gate with an 11:1 ratio into each 384-well plate.
Gating strategy	Intact nuclei were first discriminated from debris by virtue of their bright DNA labeling (Hoechst Height signal) followed by light scattering profiles (Forward Scatter (FSC) Height vs Side Scatter (SSC) Height). Events with high Pulse Width measurements for FSC and SSC were then excluded as aggregates. Next, NeuN-AlexaFluor 488 positive or negative nuclei were selected, reaching an 11:1 ratio of NeuN+ to NeuN- nuclei.

☒ Tick this box to confirm that a figure exemplifying the gating strategy is provided in the Supplementary Information.
